# Accuracy of ultrasound estimation of fetal weight in twin pregnancy

**DOI:** 10.1002/ijgo.16186

**Published:** 2025-02-24

**Authors:** Patrick Dicker, Ronan Conroy, Fionnuala McAuliffe, Michael Geary, Sean Daly, John J. Morrison, Stephen Carroll, Fergal D. Malone, Fionnuala M. Breathnach

**Affiliations:** ^1^ Royal College of Surgeons in Ireland, Obstetrics and Gynecology Dublin Ireland; ^2^ Department of Epidemiology and Public Health Royal College of Surgeons in Ireland Dublin Ireland; ^3^ UCD Perinatal Research Center University College Dublin Dublin Ireland; ^4^ National Maternity Hospital Dublin Ireland; ^5^ Rotunda Hospital Dublin Ireland; ^6^ Department of Obstetrics and Gynecology University of Galway Galway Ireland; ^7^ University Hospital Galway Galway Ireland

**Keywords:** estimated fetal weight, fetal growth, twin gestation

## Abstract

**Objective:**

To determine the accuracy of formulas for estimation of fetal weight in twin pregnancy.

**Methods:**

Inclusion criteria from the ESPRiT twin cohort were twin pregnancies that resulted in live‐born twins without congenital anomalies or twin‐twin transfusion syndrome, ultrasound examination within 3 days of delivery, birth weight (BW) > 500 g and gestational age > 24 weeks. A total of 63 formulas using various combinations of abdominal circumference (AC), femur length (FL), biparietal diameter (BPD) and head circumference (HC) for the estimation of fetal weight (EFW), were compared to BW for accuracy in 226 twin pregnancies/452 fetuses.

**Results:**

Using median percentage error (MPE), the most accurate formulas were the Hadlock formulas (1984, 1985 with MPE < 1%) incorporating AC, HC, and FL. The INTERGROWTH‐21st formula, which incorporates AC and HC, marginally underestimated BW (MPE −3.7%). The Hadlock formulas were more likely to overestimate BW than underestimate it. Using small‐for‐gestational (SGA, EFW < 5th centile), the sensitivity for detection of low BW (BW < 5th centile) was 92%, 81% and 89% for the Hadlock 1984, Hadlock 1985 and INTERGROWTH‐21st formulas, respectively. SGA prevalence was noted to be higher for the INTERGROWTH‐21st formula for fetal factors.

**Conclusion:**

Among 63 proposed formulas for EFW, the Hadlock formulas incorporating HC, AC and FL were the most accurate in twins. However, the INTERGROWTH‐21st standard, which incorporates AC and HC, did not show lower detection rates for LBW. The frequency and intensity of twin ultrasound surveillance is such that restriction of ultrasound examination to two parameters in ultrasound could be recommended without diminution of accuracy.

## Synopsis

Our study suggests the INTERGROWTH‐21st fetal weight formula, which incorporates abdominal circumference and head circumference, can be recommended without a loss in accuracy.

## 1 INTRODUCTION

Numerous formulas have been proposed for the estimation of fetal weight (EFW) in utero. EFW is a proxy for fetal size and, using birth weights as a basis for comparison, formulas have been developed using various combinations of measurements of fetal abdominal circumference (AC), head circumference (HC), femur length (FL) and biparietal diameter (BPD). Nevertheless, in practice, an EFW is interpreted as the actual in utero weight of a fetus. Owing to reduced growth velocity in the third trimester, and lower birth weights relative to singleton pregnancies,^1^, ^2^, ^3^, ^4^ accurate estimation of fetal weight in twins is desirable, particularly in the context of high rates of iatrogenic and spontaneous preterm delivery and evaluation for inter‐twin growth discordance. Serial determination of fetal weight is recommended by NICE,^5^ ISUOG,^6^ ACOG,^7^ RCOG^8^, ^9^ throughout pregnancy for both monochorionic and dichorionic twin gestations.

In the context of twin pregnancy, comparative studies have used a limited number of EFW formulas, without consensus on the most accurate formula.^10^, ^11^, ^12^, ^13^, ^14^ These studies were performed prior to development of the INTERGROWTH‐21st estimated fetal weight standard.^15^ In this study, we sought to compare the accuracy of a comprehensive set of fetal weight formulas,^10^, ^11^, ^12^, ^13^, ^14^, ^15^, ^16^, ^17^, ^18^, ^19^, ^20^, ^21^, ^22^, ^23^, ^24^, ^25^, ^26^, ^27^, ^28^, ^29^, ^30^, ^31^, ^32^, ^33^, ^34^, ^35^, ^36^, ^37^, ^38^, ^39^, ^40^, ^41^, ^42^, ^43^, ^44^, ^45^, ^46^, ^47^, ^48^, ^49^, ^50^, ^51^, ^52^, ^53^, ^54^, ^55^ including formulas developed specifically for twin gestations.^13^, ^14^ We also sought to determine if a formula with optimal accuracy performs better than existing formulas, recommended for singletons, in the detection of low birth weight (LBW) for gestational age and for significant birth weight discordance.

## 2 MATERIALS AND METHODS

The prospective evaluation of sonographic predictors of restricted growth in twins study (ESPRiT Study^53^, ^54^, ^55^) was conducted at eight academic perinatal centers in Ireland, all with tertiary neonatal intensive care facilities. Institutional review board approval was obtained at each participating site and the study participants gave written informed consent. Inclusion criteria were all twin pregnancies presenting to the study centers between 11 and 22 completed weeks of gestation, with both fetuses alive at the time of enrolment, and with intact membranes. Monoamnionicity, a major structural abnormality in either twin, or fetal aneuploidy (either suspected or confirmed) led to exclusion from the study.

All patients who met the inclusion criteria underwent a program of intensive fetal surveillance carried out by dedicated research ultrasonographers using standardized ultrasonographic equipment (GE Voluson Expert 730; GE Healthcare Ultrasound, Milwaukee, Wisconsin). Chorionicity was assigned by standard ultrasonographic criteria at the first ultrasonographic evaluation and confirmed with placental pathology. A study‐specific pregnancy dating protocol was implemented across all centers.

Two‐weekly growth scans were performed from 16 weeks of gestation until delivery for monochorionic twin pairs and from 24 weeks until delivery in dichorionic pregnancies. Standard fetal biometry was recorded (abdominal circumference, biparietal diameter, head circumference, femur length). Biparietal diameter was assessed inner‐to‐outer and abdominal circumference and head circumference were assessed using the ellipse method. The Hadlock 4‐parameter formula (1985), which incorporates biparietal diameter, was used to determine estimated fetal weight, with other formulas used when a fetal biometry parameter was not available.^22^ A quality review system was in place which required weekly submission by ultrasonographers of images to a central ultrasonography quality assurance committee. All prenatal and ultrasonographic data were contemporaneously transferred to an ultrasonography software system (Viewpoint; MDI Viewpoint, Jacksonville, Florida), uploaded onto a live web‐based central consolidated database and screened for potential anomalous findings. Database quality reports were generated prospectively at regular intervals with a feedback loop between the data management team and the study‐dedicated sonographers.

### 2.1 Study population and evaluation

From the unselected prospective cohort of twins, we selected those twins with an ultrasound examination within 3 days of delivery. We evaluated the accuracy of EFW formulas in this group using several statistical measures and for their determination of small‐for‐gestational age (SGA) status. We then evaluated the association of SGA, defined by a variety of formulas, and the observation of low birth weight (LBW) for gestational age. The prevalence of SGA in subgroups of the study population was explored.

### 2.2 Formulas for comparison

We took a similar set of formulas considered by Hammami et al.^56^ in their assessment of singleton pregnancies, excluding those formulas targeting large‐for‐gestational age fetuses and pregnancies complicated by diabetes. In addition, we included the following formulas:

1. Two formulas developed specifically for twins^13^, ^14^ both incorporating AC and FL alone.
2. Seven alternative formulas proposed by Hadlock et al.^22^ that is, their earlier 1984 suite of formulas. To avoid potential confusion, the complete suite of formulas are presented in Table 1, with labels “A” and “B”. The Hadlock formula incorporating HC, AC and FL from the subsequent 1985 publication,^23^ formula B3, is commonly applied in singleton pregnancies.
3. The volumetric formulas of Shinozuka^39^, ^40^ and Jackson et al.^30^ which include BPD for determination of EFW.

**TABLE 1:** The Hadlock suite of formulas from 1984^22^ and 1985.^23^

Label^a^	Biometry used	Formula
1984 publication		
A1	AC	Log_e_ BW = 2.695 + 0.253 × AC − 0.00275 × AC^2^
A2	AC, BPD	Log_10_ BW = 1.1134 + 0.05845 × AC − 0.000604 × AC^2^ − 0.007365 × BPD^2^ + 0.000595 × BPD × AC + 0.1694 × BPD
A3	AC, HC	Log_10_ BW = 1.182 + 0.0273 × HC + 0.07057 × AC − 0.00063 × AC^2^ − 0.0002184 × HC × AC
A4	AC, FL	Log_10_ BW = 1.3598 + 0.051 × AC + 0.1844 × FL − 0.0037 × AC × FL
A5	AC, BPD, FL	Log_10_ BW = 1.4787 − 0.003343 × AC × FL + 0.001837 × BPD^2^ + 0.0458 × AC + 0.158 × FL
A6	AC, HC, FL	Log_10_ BW = 1.5662 − 0.0108 × HC + 0.0468 × AC + 0.171 × FL + 0.00034 × HC^2^ − 0.003685 × AC × FL
A7	AC, HC, BPD, FL	Log_10_ BW = 1.5115 + 0.0436 × AC + 0.1517 × FL − 0.00321 × AC × FL + 0.0006923 × BPD × HC
1985 publication		
B1	AC, FL	Log_10_ BW = 1.304 + 0.05281 × AC + 0.1938 × FL − 0.004 × AC × FL
B2	AC, BPD, FL	Log_10_ BW = 1.335 + 0.0316 × BPD + 0.0457 × AC + 0.1623 × FL − 0.0034 × AC × FL
B3	AC, HC, FL	Log_10_ BW = 1.326 − 0.00326 × AC × FL + 0.0107 × HC + 0.0438 × AC + 0.158 × FL
B4	AC, HC, BPD, FL	Log_10_ BW = 1.3596 + 0.0064 × HC + 0.0424 × AC + 0.174 × FL + 0.00061 × BPD × AC − 0.00386 × AC × FL

Abbreviations: AC, abdominal circumference; BPD, biparietal diameter; BW, birth weight; FL, femur length; HC, head circumference.

^a^ The labeling A1–A7 and B1–B4 is used here is for clarity in the manuscript. It is assumed that formula A1 had a typographical error in the original publication and the natural log, not log base 10, is correct.

A total of 63 EFW formulas^13^, ^14^, ^15^, ^16^, ^17^, ^18^, ^19^, ^20^, ^21^, ^22^, ^23^, ^24^, ^25^, ^26^, ^27^, ^28^, ^29^, ^30^, ^31^, ^32^, ^33^, ^34^, ^35^, ^36^, ^37^, ^38^, ^39^, ^40^, ^41^, ^42^, ^43^, ^44^, ^45^, ^46^, ^47^, ^48^, ^49^, ^50^, ^51^, ^52^ were compared. The required biometry inputs (in cm units) and generic code for all the formulas are provided as Appendix S1. Three key formulas are presented for comparison in the tables: the Hadlock B3 formula (commonly applied in singletons, which uses AC, HC and FL), the Stirnemann et al.^15^ formula (the INTERGROWTH‐21st standard, which uses AC and HC) and the Hadlock B4 formula (used prospectively in this study population and uses all four biometry parameters).

### 2.3 Measures of accuracy

To determine the proximity of the EFW determinations to birth weight, comparisons were made using percentage error (PE):

$$\text{Percentage error}\;\left(\mathrm{PE}\right)=100\%\times \frac{\left(\mathrm{EFW}-\mathrm{BW}\right)}{\mathrm{BW}}$$

Median PE was used to rank the formulas from “most accurate” to “least accurate”. However, the percentage within 10% of birth weight (|PE| ≤ 10%) was also considered as a meaningful summary as this quantifies variability in PE. Formulas were then compared for agreement using McNemar's test for paired data. We assessed whether PE indicated overestimation of BW (PE > 10%) or underestimation of BW (PE < −10%) and summarized these results as a ratio.

Using the INTERGROWTH‐21st fetal growth chart standard for singletons,^57^ we defined small‐for‐gestational age (SGA) as EFW < 5th centile for each formula. The fifth centile was chosen as this is the lowest available centile from the INTERGROWTH‐21st standard.

### 2.4 Perinatal outcomes and subgroup comparisons

With SGA defined using the different EFW formulas, we sought to determine their predictive accuracy for low birth weight (LBW), defined as birth weight < 5th centile using the WHO‐UK standard.^58^, ^59^ The INTERGROWTH‐21st standard for birth weight^60^ was not considered because this reference range begins at 33 weeks' gestation. Predictive accuracy for birth weight discordance of ≥18% was also evaluated. In this study population, the 18% discordance in BW was found to be an independent predictor of adverse perinatal outcome.^53^ The sensitivity and false‐positive rate for the detection of LBW and BW discordance ≥18% was examined.

A comparative analysis of the most accurate formula and the three key formulas were examined for prevalence of SGA. The following fetal factors were considered in this analysis: chorionicity; fetal presentation; baby gender and HC/AC > 1.1. The cutoff of 1.1 for the HC/AC ratio was determined from the study by Grantz et al.^2^ as a potential indicator of growth disproportionality.

## 3 RESULTS

Among 1028 enrolled twin pregnancies, there were 15 congenital anomalies, 24 twin pairs with a previable single or dual death (gestational age at delivery <24 weeks or birth weight <500 g), 16 cases of twin‐twin transfusion syndrome (TTTS), eight pregnancies that delivered outside of a participating study center and 17 participants withdrew from the study. These cases were excluded from further analysis and, with some overlap in these criteria, resulted in 948 twin‐pairs for further evaluation. Of these, 226 twin‐pairs (452 fetuses) had an ultrasound examination within 3 days of delivery.

The median [IQR] maternal age was 33 [27, 39] years and the ethnicity of the study participants was primarily Caucasian (88%) in this subcohort. Body mass index (BMI, calculated as weight in kilograms divided by the square of height in meters) in excess of 30 at enrolment occurred in 15% of participants and 51% of participants were nulliparous. Monochorionic and dichorionic twins represented 23% and 77% of pregnancies, respectively. The rate of preterm delivery was 19% and the rate of SGA (EFW < 5th centile using the singleton standard) was 11% (Table 2).

**TABLE 2:** Pregnancy characteristics of twins delivering within 3 days of last ultrasound examination.

Characteristic	Summary
**Twin‐pairs**	***N* = 226**
Maternal age (years)	33 [27, 39]
BMI ≥30 kg/m^2^	34 (15%)
Nulliparity	113 (51%)
Chorionicity	
Monochorionic	52 (23%)
Dichorionionic	174 (77%)
GA at delivery (week)	36.3 [32.0, 38.0]
Preterm delivery (<34 weeks)	42 (19%)
BW discordance ≥18%^a^	73 (32%)
GA at last examination (week)	36.1 [31.7, 37.7]
**Singular twins**	***N* = 452**
Birth weight (grams)	2440 [1490, 3080]
Low BW (<5th centile)^b^	48 (11%)
Sex	
Female	234 (52%)
Male	216 (48%)
Fetal presentation at last examination	
Breech	105 (28%)
Transverse	57 (15%)
Cephalic	212 (57%)

*Note*: BMI, calculated as weight in kilograms divided by the square of height in meters.

Abbreviations: BMI, body mass index; BW, birth weight; GA, gestational age.

^a^ Based on the optimal cutoff found in the full study cohort.^53^

^b^ Defined according to the WHO‐UK gender‐specific reference range.^58^, ^59^

**FIGURE 1:**
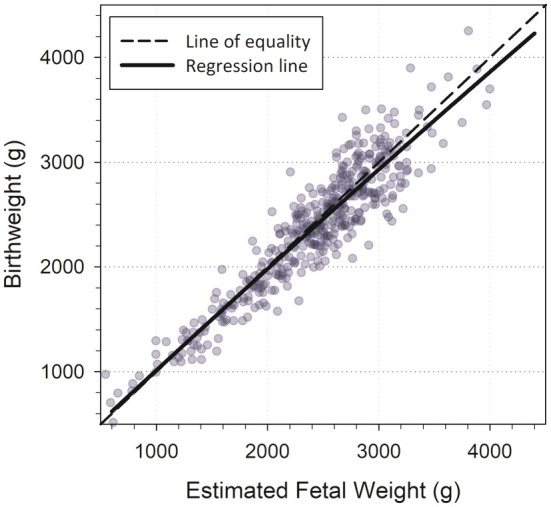
Scatterplot of birth weight versus estimated fetal weight at last examination within 3 days of delivery (*N* = 452).

A comparison of birth weights and estimated fetal weights in those with an ultrasound examination within 3 days of delivery is shown in Figure 1. EFW, as assessed using the Hadlock B4 and other formulas during the study, overestimates birth weights by a small degree on average (regression line) and this overestimation increases as birth weight levels increase in the study population.

### 3.1 Comparison of fetal weight formulas

The full set of accuracy results, across all formulas considered, are provided as Appendix S1. Table 3 presents a select set of key comparisons from the 63 EFW formulas. The most accurate formula, as judged by median percentage error (MPE), is the earlier published Hadlock 3‐parameter formula that uses HC, AC and FL (Formula A6, Table 1).^22^ The MPE is −0.5%, indicating that this formula underestimates birth weight by a small percentage across the whole study population. A total of 69% of fetuses were within 10% of birth weight, with overestimation (PE > 10%) and underestimation (PE < −10%) of birth weight reasonably well balanced (17% and 14%, respectively). The percentage considered SGA < 5th centile was 15%.

**TABLE 3:** Accuracy of selected formulas for estimation of fetal weight in twins.

Formula	Biometry used	MPE	|PE| ≤ 10%		PE > 10% (a)	PE < −10% (b)	a/b	SGA (<5th centile)^b^
		%	%	*P* value^a^	%	%	Ratio	%
Most accurate formula								
Hadlock A6^22^	AC, HC, FL	−0.5	69	Ref	17	14	1.2	15
Least accurate formula								
Kohorn^33^	BPD	13.8	35	<0.001	60	5	12.0	1
Other key formulas								
Hadlock B3^23^	AC, HC, FL	1.0	68	0.465	21	11	1.9	13
Stirnemann et al.^33^	AC, HC	−3.7	66	0.192	13	21	0.6	16
Hadlock B4^23^	AC, HC, BPD, FL	1.8	69	0.879	22	9	2.5	13

Abbreviations: AC, abdominal circumference; BPD, biparietal diameter; FL, femur length; HC, head circumference; MPE, median percentage error; PE, percentage error; SGA, small‐for‐gestational age.

^a^ McNemar's test for paired data with Hadlock A6 used as a reference comparison.

^b^ Defined using the INTERGROWTH‐21st standard (Kiserud et al.^57^).

The formula identified as least accurate was that of Kohorn^33^ and this finding was consistent across all the accuracy measures. The percentage within 10% of birth weight was 35% and this was statistically significantly (*P* < 0.001) when compared to the Hadlock A6 formula.

Two key comparisons were those of the Hadlock B4 formula (used in this study) and the Hadlock B3 formula (commonly used for singletons). Both of these formulas originate from the later 1985 paper by Hadlock et al.^23^ These show similar MPE, indicating a marginal overall overestimation of birth weight (1.8% and 1.0%, respectively) overall. These formulas also had similar percentages within 10% of birth weight (68% and 69%, respectively), not statistically significant when compared to the most accurate formula (Hadlock A6). However, overestimation (in excess of +10%) was approximately twice as likely to occur than underestimation (<−10%) with these formulas. This imbalance is reflected in the ratio of overestimation to underestimation, 2.5 and 1.9, for Hadlock B4 and B3, respectively. The percentage defined as SGA using these formulas were similar to the Hadlock B6 formula.

The INTERGROWTH‐21st formula (Stirnemann et al.^15^) was also included as a key comparator. The MPE was −3.7% and the percentage within 10% of birth weight was 66%, not statistically significant when compared to the most accurate formula. In contrast to the Hadlock formulas, this formula was almost twice as likely to underestimate rather than overestimate birth weights (ratio = 0.6), for twin examinations within 3 days of delivery. The percentage defined as SGA was 16% using this formula.

Using SGA defined for each formula as presented in Table 3, the detection rates for LBW (BW <5th centile) and significant birth weight discordance (≥18%) are presented in Table 4. The least accurate formula, that of Kohorn,^33^ was excluded from this analysis. All formulas exhibited high detection rates for LBW (>80%), with the Hadlock A6 and Stirnemann^15^ formulas having better sensitivity (92% and 89%, respectively) than the other formulas. The false positive rates (SGA percent for BW ≥5%) were higher for Hadlock A6 and Stirnemann^15^ formulas, 17% and 25%, respectively.

**TABLE 4:** Percent SGA (EFW < 5th centile) according to perinatal outcome and study subgroup.

Outcome	Subgroup	*N*	Hadlock et al. B3^23^ (%)	Hadlock et al. A6^22^ (%)	Stirnemann et al.^15^ (%)	Hadlock et al. B4^23^ (%)
LBW	BW < 5th centile	48	81	92	89	86
	BW ≥ 5th centile	402	14	17**	24***	14
BW discordance	≥18%	144	30	34	42**	32
	<18%	306	16	20**	25***	15
Chorionicity	Monochorionic	102	18	21	28*	18
	Dichorionic	348	21	26**	31***	21
Presenting twin	Presenting twin	226	23	26	31**	22
	Non‐presenting twin	224	18	23*	29***	20
Sex	Female	215	27	31*	33*	27
	Male	234	14	19*	28***	15
Presentation at last ultrasound	Breech	105	23	26	27	24
	Transverse	57	25	30	32	27
	Cephalic	212	20	25*	33***	19
HC/AC ratio	>1.1	61	49	54	70**	51
	≤1.1	389	15	19**	23***	15

*Note*: Row percents are presented in the table. McNemar's test was used to compare SGA rates between formulas, with the Hadlock et al. B3 formula as a reference: *P value <0.05, **P value <0.01, ***P value <0.001.

Abbreviations: BW, birth weight; EFW, estimated fetal weight; HC/AC ratio, head circumference to abdominal circumference ratio; LBW, low birth weight; SGA, small for gestational age.

Similar results are found when considering BW discordance ≥18% as an outcome. However, the Stirnemann^15^ formula has a higher detection rate (42%) when compared to the Hadlock A6 formula (34%). Fetal factors that may influence fetal weight estimation are also presented in Table 4.

In fetuses differing in proportionality, as illustrated by the HC/AC ratio, there was a significant disparity in SGA prevalence rates between the Stirnemann^15^ formula and the Hadlock formulas. In those with HC/AC > 1.1, the Stirnemann formula^15^ identified 70% as SGA, compared to approximately 50% for the Hadlock formulas.

## 4 DISCUSSION

Hadlock et al.^22^, ^23^ proposed several EFW formulas based on the availability of fetal biometric parameters. The applicability of Hadlock's formulas has been validated in diverse populations, from Rio de Janeiro^61^ to the eastern regions of Nepal.^62^ The INTERGROWTH‐21st standard for estimation of fetal weight^15^ was conducted with a strong ultrasound quality protocol for singleton pregnancies in six countries, bolstered by a comprehensive and robust statistical evaluation. It has been recommended as a prescriptive international standard to complement the WHO Child Growth Standards.^63^ This formula uses abdominal circumference and head circumference alone in the determination of fetal weight.

Several comparative studies^10^, ^11^, ^12^, ^13^, ^14^ of formulas for estimation of fetal weight in twins have been performed on a limited selection of formulas, prior to the introduction of the fetal weight formula by the INTERGROWTH‐21st consortium.

### 4.1 Summary of main findings

Our results suggest that the Hadlock A6 formula, which uses AC, HC and FL, performs better than the Hadlock B3 formula, which uses the same biometry parameters, in twin pregnancy. The same formula was identified by Khalil et al.^12^ as providing the most accurate representation of fetal size in twins.

Our results also indicate that the INTERGROWTH‐21st formula did not have a lower detection rate for LBW and performed better for detection of significant birth weight discordance than the Hadlock A6 or Hadlock B3 formulas. Where significance discordance in AC to HC might be evident, the INTERGROWTH‐21st formula is potentially a better alternative than the Hadlock formulas. The overall small degree of underestimation of birth weight might be ascribed to growth in the 3 days from ultrasound examination to delivery.

The limitations of our study are the small sample size of twins having an ultrasound within 3 days of delivery and the relative homogeneity of ethnicity in the study population. Strengths of the study were the prospective recruitment, assessment of fetal biometry and ascertainment of outcomes in an unselected population and the comprehensive set of EFW formulas considered for comparison, including the INTERGROWTH‐21st standard.

### 4.2 Implications for further research

In the context of research in twins, the Hadlock B3 formula for estimation of fetal weights seems well‐established. This formula has been used to determine references ranges in large twin populations,^64^, ^65^ to determine customized centiles in twins^66^ and has been combined with birth weights to determine a birth weight reference that seeks avoid biases associated with preterm delivery.^67^ Nevertheless, given the considerable overestimation relative to underestimation of birth weights in twins using the Hadlock B3 formula, it seems that the INTERGROWTH‐21st formula merits consideration for use in twin gestations. Further studies evaluating EFW formulas in more ethnically diverse populations, and the potential influence of chorionicity, may add to the evidence provided by our study.

## 5 CONCLUSION

We have identified that among 63 published formulas for the estimation of fetal weight, one of Hadlock's 1984 models (incorporating HC, AC and FL) performed better than the commonly used 1985 formula, in the prediction of birth weight in a prospectively recruited twin population. Our results also indicate that the INTERGROWTH‐21 standard should be explored further in twin gestations. In this study, the INTERGROWTH‐21st standard, which incorporates AC and HC, did not show lower detection rates for LBW. The frequency and intensity of twin ultrasound surveillance is such that restriction of ultrasound examination to two parameters in ultrasound could be recommended without a reduction in accuracy.

## AUTHOR CONTRIBUTIONS

Patrick Dicker was responsible for data management, statistical analysis and manuscript writing. All other authors (Ronan Conroy, Fionnuala McAuliffe, Michael Geary, Sean Daly, John J. Morrison, Stephen Carroll, Fergal D. Malone, Fionnuala M. Breathnach) recruited patients for the study and/or provided input to manuscript preparation.

## FUNDING INFORMATION

This study (secondary analysis) was conducted without funding.

## CONFLICT OF INTEREST STATEMENT

The authors have no conflicts of interest.

## Supporting information


Appendix S1

## Data Availability

Research data are not shared.
